# Tumor suppressive role of microRNA-4731-5p in breast cancer through reduction of PAICS-induced FAK phosphorylation

**DOI:** 10.1038/s41420-022-00938-1

**Published:** 2022-04-04

**Authors:** Lei Lang, Jing Tao, Chaomei Yang, Wei Li

**Affiliations:** 1 Department of Clinical Laboratory, Chongqing Emergency Medical Center, Chongqing University Central Hospital, School of Medicine, Chongqing University, Chongqing, 400014 P. R. China; 2 Department of Rheumatology and Immunology, Children’s Hospital of Chongqing Medical University, Chongqing, 400016 P. R. China

**Keywords:** Breast cancer, Breast cancer

## Abstract

A wide array of microRNAs (miRNAs) is differentially expressed in breast tumors and also functions as tumor suppressors. Herein, the current study sought to unravel the function of miR-4731-5p in breast cancer progression. First, breast cancer-related miRNA and mRNA microarray data sets were retrieved for differential analyses. Subsequently, the expression patterns of miR-4731-5p, PAICS, and FAK in breast cancer tissues and cells were determined, in addition to analyses of their roles in glycometabolism, migration, invasion, epithelial–mesenchymal transition (EMT) analyzed through functional assays. Next, the targeting relation between miR-4731-5p and PAICS was validated. Xenograft tumors in nude mice were further established to reproduce and verify the in vitro findings. miR-4731-5p was poorly expressed and PAICS was highly expressed in breast cancer tissues and cells. PAICS was confirmed as a target of miR-4731-5p. Moreover, miR-4731-5p exerted an inhibitory effect on glycolysis, EMT, migration, and invasion in breast cancer cells via regulation of PAICS-dependent phosphorylation of FAK. In vivo assay further validated the significance of the miR-4731-5p/PAICS/FAK axis in vivo tumorigenesis and lung metastasis in breast cancer. Collectively, our findings indicated that miR-4731-5p inhibited breast cancer cell glycolysis and EMT through the reduction of PAICS-induced phosphorylation of FAK.

## Introduction

Breast cancer is one of the most common malignancies in the female population, and also represents the leading cause of cancer-related deaths among women [1]. Despite the tremendous advancements in regard to the laboratory, epidemiological and clinical investigations, the morbidity of breast cancer continues to grow [2]. Advanced-stage breast cancer manifests in the form of distant organ metastases, which remain untreatable using currently available strategies [3]. Epithelial–mesenchymal transition (EMT) represents a crucial process in malignancies, wherein epithelial cells transform into a mesenchymal phenotype with enhanced invasive ability to distant sites, thus contributing to tumor progression and metastasis [4]. Meanwhile, the past decade has witnessed a surge of microRNAs (miRNAs) in crucial processes such as tumorigenesis and tumor metastasis, with certain miRNAs implicated in the regulation of tumor invasion/metastasis *via* EMT- and/or non-EMT-dependent mechanisms [5]. In lieu of the same, it would be practical to investigate the miRNA-mediated EMT mechanism in regard to breast cancer to aid the development of therapeutic targets against the lethal malignancy.

Inherently, miRNAs are defined as short, non-coding RNAs, which possess the ability to repress the translation of target messenger RNA (mRNA)s and induce their degradation [6]. One such miRNA, miR-4731-5p (miR-4731) is regarded as a melanoma-enriched miRNA, whereas its deficiency is known to contribute to the progression of advanced melanomas [7]. Interestingly, miR-4731-5p has been previously highlighted as a tumor-suppressor in glioma [8], especially in glioblastoma multiforme [9]. Meanwhile, the PAICS gene was recently indicated as a tumor-promotive gene in several human cancers including lung adenocarcinoma (LADC) [10], cervical cancer [11], and even breast cancer [12]. Moreover, PAICS is capable of allosterically-activating pyruvate kinase M2 (PKM2), which directly binds to integrin β1 and consequently activates focal adhesion kinase (FAK) [13, 14]. The latter is noteworthy as FAK is established as a crucial inducer of growth factors and anchorages, and also a promoter of glycolysis through upregulation of pivotal glycolytic proteins, such as enolase and PKM2 [15]. Interestingly, a prior study unveiled that the loss of FAK in cancer-associated fibroblasts (CAFs) could enhance chemokine production, and subsequently promote malignant cell glycolysis [16]. In addition, FAK activation underlies IGF1R-dependent EMT, migration, and invasion in triple-negative breast cancer [17]. As PAICS was highlighted as a potential target gene of miR-4731-5p by initial bioinformatics analysis in our investigation, we speculated that miR-4731-5p might regulate the oncogene PAICS to modulate FAK-driven glycolysis and EMT in breast cancer. Accordingly, we carried out a series of cellular and animal experiments to testify this hypothesis, in an effort to broaden our understanding of breast cancer progression.

## Results

### miR-4731-5p and PAICS are predicted to be involved in breast cancer

**Fig. 1:**
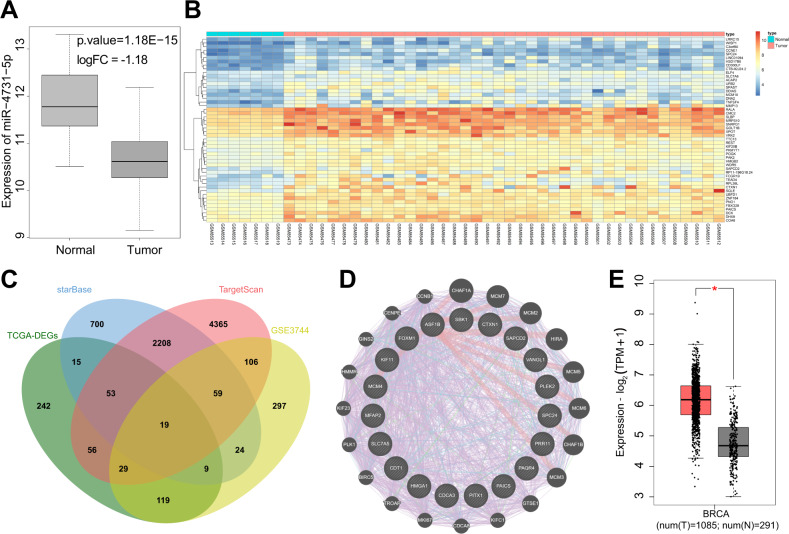
Analysis of miR-4731-5p expression in microarray data set and prediction of downstream regulatory target genes. **A** The differential expression of miR-4731-5p in GSE57897, the *x* axis represents the sample type, the *y* axis represents the expression value, the left box plot represents normal samples, the right box plot represents tumor samples, and the upper right is the differential *p* value. **B** Heat maps of the top 50 significantly upregulated gene (arranged in positive sequence based on an adjusted *p* value) in GSE3744 data set including seven normal samples and 40 tumor samples. The *x* axis represents the sample number, the *y* axis represents the gene name, and the tree diagram on the left represents the gene expression level clustering (adjusted *p* value). Each small square in the figure represents the expression of a differential gene in a sample, the histogram on the upper right is the color scale. **C** Prediction of downstream target genes of miR-4731-5p. The four ellipses in the figure are the significantly high expression genes in breast cancer retrieved by TCGA, the target genes predicted by starBase and TargetScan databases, and the significantly high expression genes in the GSE3744. The central part is the intersection of four sets of data. **D** Interaction analysis of candidate target genes, the middle part of the figure represents 19 candidate genes, the outer circle represents 20 genes that are related to the candidate gene, and the line between the circles indicates that there is an association between genes. **E** The differential expression of PAICS genes in breast cancer and normal samples included in TCGA and GTEx. The *x* axis indicates the sample type, the *y* axis indicates the expression value, the red box plot indicates the tumor sample, and the gray box plot indicates the normal sample. *p < 0.01.

Initial analyses of the GSE57897 microarray data set indicated a reduction in miR-4731-5p expression in breast cancer samples (Fig. 1A), however, there is no relevant research in regard to the role of miR-4731-5p in breast cancer. In order to further elucidate the mechanism of miR-4731-5p in breast cancer, the target genes of miR-4731-5p were predicted using the starBase and TargetScan databases. Additionally, the significantly highly expressed genes in TCGA breast cancer were retrieved from the GEPIA2 database. In addition, 662 significantly highly expressed genes were obtained from the microarray data set GSE3744 (Fig. 1B). Subsequently, the predicted target genes from starBase and TargetScan, highly expressed genes in TCGA, and highly expressed genes from the GSE3744 data set were intersected, obtaining a total of 19 intersected genes (Fig. 1C). Further interaction analyses of these 19 genes were carried out, and their related genes were amplified at the same time to obtain the interaction score, with PAICS exhibiting the highest score in the interaction network (Fig. 1D and Supplementary Table 2).

Further quantitative analysis of the top 10 genes (namely, SBK1, CTXN1, SAPCD2, VANGL1, PLEK2, SPC24, PRR11, PAQR4, PAICS, and PITX1) with the highest interaction scores validated that the difference in PAICS expression was the most pronounced (Supplementary Fig. 1). Moreover, PAICS was also significantly highly expressed in breast cancer tissue samples included in the TCGA and GTEx databases (Fig. 1E). Meanwhile, existing evidence indicates that PAICS possesses the ability to regulate the progress of glycolysis by regulating the phosphorylation of FAK [14]. Altogether, these findings highlight the involvement of miR-4731-5p and PAICS in breast cancer.

### miR-4731-5p suppresses glycolysis, EMT, migration, and invasion of breast cancer cells

**Fig. 2:**
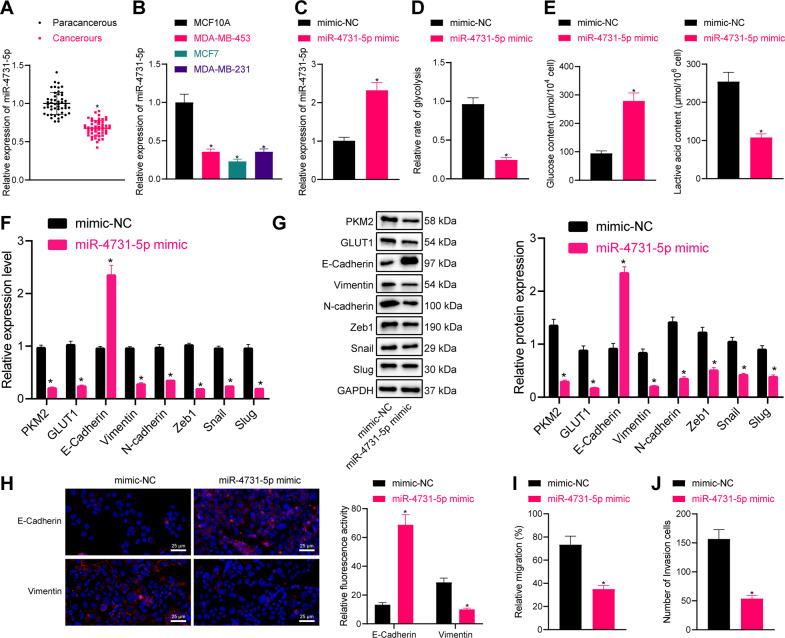
miR-4731-5p suppresses the glycolysis, EMT, and migration and invasion of MCF-7 cells. **A** RT-qPCR was used to detect the expression of miR-4731-5p in clinical samples (*n* = 50). **B** RT-qPCR was used to detect the expression of miR-4731-5p in breast cancer cells (MDA-MB-453, MCF-7, and MDA-MB-231) and breast epithelial cells. **C** RT-qPCR was used to detect the expression of miR-4731-5p after miR-4731-5p mimic treatment. **D** The effect of miR-4731-5p on glycolysis of breast cancer cells. **E** The effect of miR-4731-5p on glucose and lactic acid content of breast cancer cells. **F** RT-qPCR was used to detect the expression of glycolysis-related genes PKM2, GLUT1, and EMT-related markers Vimentin and E-Cadherin. **G** Western blot analysis was used to detect the expression of glycolysis-related genes PKM2, GLUT1, and EMT-related markers Vimentin and E-Cadherin. **H** Immunofluorescence was used to detect the expression of EMT-related markers Vimentin and E-Cadherin, scale bar = 25 μm. **I** Detection of cell migration by scratch test after miR-4731-5p mimic treatment. **J** Detection of cell invasion by Transwell assay after miR-4731-5p mimic treatment. Measurement data were expressed as mean ± standard deviation. Comparison between two groups was performed through an independent *t* test. Analysis among multiple groups was conducted by one-way ANOVA followed by Tukey’s post hoc test. * *p* < 0.05 compared with adjacent normal tissues, MCF10A cell, or mimic NC treatment.

miR-4731-5p has been previously shown to suppress tumor metastasis in malignant tumors, but its role in breast cancer remains elusive [8]. The results of RT-PCR demonstrated that miR-4731-5p expression levels were diminished in breast cancer tissues (Fig. 2A and Supplementary Fig. 2A), as well as in breast cancer cells MDA-MB-453, MCF-7, and MDA-MB-231. The MCF-7 and MDA-MB-231 cell lines exhibited the least reduced miR-4731-5p expressions among the four cell lines and thus were selected for further in vitro cell experiments (Fig. 2B and Supplementary Fig. 2B).

Subsequent results from RT-qPCR also illustrated that miR-4731-5p mimic treatment brought about increased miR-4731-5p expression levels (Fig. 2C, Supplementary Fig. 3A). In addition, miR-4731-5p mimic treatment reduced the glycolysis rate, glucose consumption, and lactic acid content (Fig. 2D, E and Supplementary Fig. 3B, C). Moreover, increased miR-4731-5p led to reduced expressions of glycolysis-related genes PKM2, GLUT1, and mesenchymal marker Vimentin, while increasing those of the epithelial-related marker E-Cadherin (Fig. 2F and Supplementary Fig. 3D). Western blot assay results were in line with those of RT-qPCR (Fig. 2G and Supplementary Fig. 3E). Immunofluorescence results demonstrated the presence of upregulated E-Cadherin, and downregulated Vimentin expressions after miR-4731-5p mimic treatment, indicating that miR-4731-5p could inhibit EMT of tumor cells (Fig. 2H and Supplementary Fig. 3F). Moreover, the cell migration and invasion abilities were found to be inhibited after overexpression of miR-4731-5p (Fig. 2I, J and Supplementary Fig. 3G, H).

Thereafter, we transfected negative control (NC) inhibitor or miR-4731-5p inhibitor into normal breast cell line MCF10A, and decreased miR-4731-5p expression levels were documented in response to miR-4731-5p inhibition (Supplementary Fig. 4A), while there were no significant differences in comparison of the above two groups in other experiments (Supplementary Fig. 4B–I), which underscored the specific effect of miR-4731-5p overexpression on glycolysis, EMT, migration, and invasion of breast cancer cells.

### MiR-4731-5p targets PAICS

**Fig. 3:**
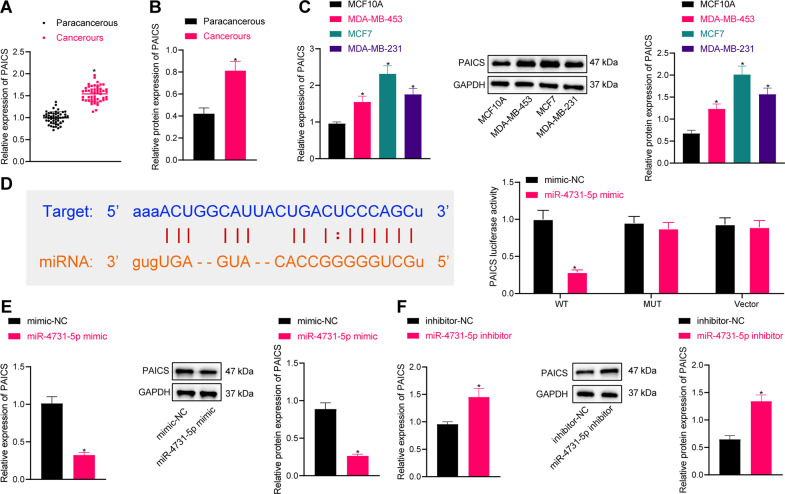
PAICS is the potential target of miR-4731-5p in MCF-7 cells. **A** RT-qPCR was used to detect the expression of PAICS in clinical samples (*n* = 50). **B** Western blot analysis was used to detect the expression of PAICS in clinical samples (*n* = 50). **C** RT-qPCR, and western blot analysis were used to detect the expression of PAICS in breast cancer cells (MDA-MB-453, MCF-7, and MDA-MB-231) and breast epithelial cell. **D** Bioinformatic analysis was used to predict the binding site of miR-4731-5p and PAICS. **E** RT-qPCR was used to detect the expression of PAICS after miR-4731-5p mimic treatment. **F** Western blot analysis was used to detect the expression of PAICS after miR-4731-5p inhibitor treatment. Measurement data were expressed as mean ± standard deviation. Comparison between two groups was performed through an independent *t* test. *p < 0.05 compared with adjacent normal tissues, MCF10A cell, mimic NC, or inhibitor-NC treatment.

PAICS was previously reported to promote the malignant progression of breast cancer [18]. Herein, the results of RT-PCR and western blot assay illustrated that PAICS expression levels were enhanced in breast cancer tissues (Fig. 3A, B). Similar elevations in PAICS expressions were observed in breast cancer cells, wherein MCF-7 and MDA-MB-231 cells exhibited the most profound upregulation, and thus were selected for further experimentation (Fig. 3C).

Subsequent in silico analysis predicted the presence of a binding site between miR-4731-5p and PAICS, which was further validated by the results of the luciferase assay (Fig. 3D). Moreover, there was a reduction in PAICS expression upon miR-4731-5p mimic treatment, while the opposing trends were documented following miR-4731-5p inhibition (Fig. 3E, F and Supplementary Fig. 5A, B). Together, the aforementioned findings indicated that miR-4731-5p could target PAICS.

### PAICS induces phosphorylation of FAK in breast cancer

**Fig. 4:**
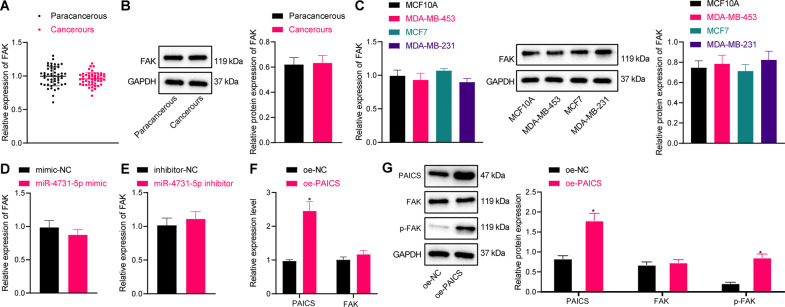
PAICS induces phosphorylation of FAK in MCF-7 cells. **A** RT-qPCR was used to detect the expression of FAK in clinical samples (*n* = 50). **B** Western blot analysis was used to detect the expression of FAK in clinical samples (*n* = 50). **C** RT-qPCR and western blot analysis were used to detect the expression of FAK in breast cancer cells (MDA-MB-453, MCF-7, and MDA-MB-231) and breast epithelial cells. **D** RT-qPCR was used to detect the expression of FAK after miR-4731-5p mimic treatment. **E** Western blot analysis was used to detect the expression of FAK after miR-4731-5p inhibitor treatment. **F** RT-qPCR was used to detect the expression of PAICS and FAK after oe-PAICS treatment. **G** Western blot analysis was used to detect the expression of PAICS, FAK, and p-FAK after oe-PAICS treatment. Measurement data were expressed as mean ± standard deviation. Comparison between two groups was performed through an independent *t* test. Analysis among multiple groups was conducted by one-way ANOVA followed by Tukey’s post hoc test. *p < 0.05 compared with adjacent normal tissues, MCF10A cell, mimic NC, inhibitor-NC, or oe-NC treatment.

Existing evidence suggests that PAICS can regulate the activity of pyruvate kinase and regulate the activity of PKM2, whereas PKM2 is regarded as a critical regulatory molecule in the glycolysis process, and can also promote the glycolysis process by phosphorylating FAK [13, 14]. Accordingly, we explored the regulatory relationship between PAICS and FAK, and uncovered that there were no significant differences in FAK expressions between breast cancer tissues and adjacent tissues (Fig. 4A, B). Moreover, we observed the same results in breast cancer cells relative to mammary epithelial cells (Fig. 4C).

Subsequent experimentation revealed that miR-4731-5p mimic/inhibitor exerted no effect on the expression of FAK (Fig. 4D, E and Supplementary Fig. 6A, B), whereas PAICS overexpression elevated PAICS expression levels, but did not influence FAK expression (Fig. 4F and Supplementary Fig. 6C). The results of Western blot analysis further illustrated that oe-PAICS treatment elevated PAICS and p-FAK expression, but exerted no influence on FAK expression levels (Fig. 4G and Supplementary Fig. 6D). Overall, these findings indicated PAICS could promote FAK phosphorylation in breast cancer cells.

### Downregulation of FAK inhibits glycolysis, EMT, migration, and invasion of breast cancer cells

**Fig. 5:**
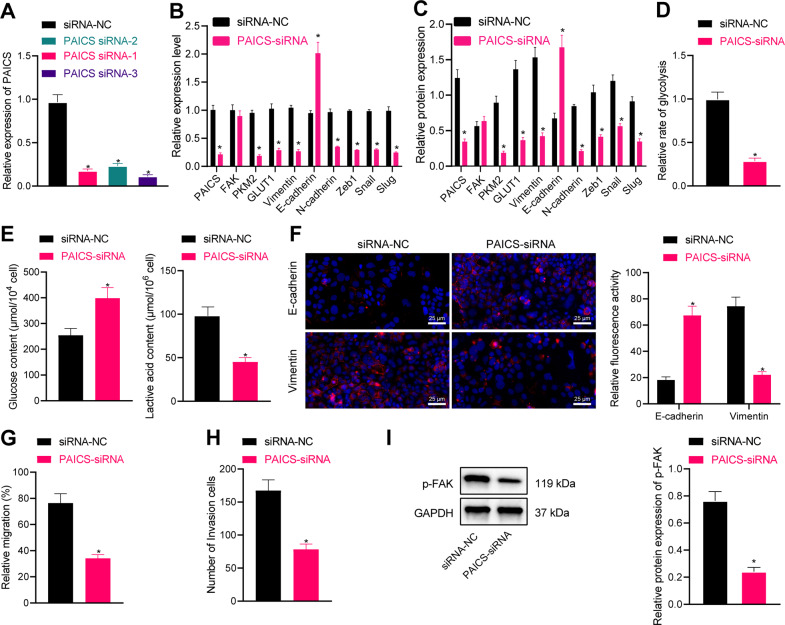
Decreased PAICS inhibits the glycolysis, EMT, migration, and invasion of MCF-7 cells. **A** RT-qPCR was used to detect the expression of PAICS in breast cancer cells. **B** RT-qPCR was used to detect the expression of PAICS, FAK, PKM2, GLUT1, Vimentin, N-cadherin, Zeb1, Snail and Slug, and E-Cadherin. **C** Western blot analysis was used to detect the expression of PAICS, FAK, PKM2, GLUT1, Vimentin, N-cadherin, Zeb1, Snail and Slug, and E-Cadherin. **D** The effect of PAICS on glycolysis of breast cancer cells. **E** The effect of PAICS on glucose and lactic acid content of breast cancer cells. **F** Immunofluorescence was used to detect the expression of EMT-related markers Vimentin and E-Cadherin, scale bar = 25 μm. **G** Detection of cell migration by scratch test after PAICS siRNA treatment. **H** Detection of cell invasion by transwell assay after PAICS siRNA treatment. **I** Western blot analysis was used to detect the expression of p-FAK after PAICS siRNA treatment. Measurement data were expressed as mean ± standard deviation. Comparison between two groups was performed through an independent *t* test. Analysis among multiple groups was conducted by one-way ANOVA followed by Tukey’s post hoc test. *p < 0.05 compared with siRNA-NC treatment.

To further elaborate on the role of PAICS in breast cancer, we first established a PAICS silencing cell line. RT-PCR results illustrated that PAICS siRNA-3 exhibited the most pronounced silencing efficiency, and thus was selected for further experiments (Fig. 5A, Supplementary Fig. 7A). Subsequent experimentation illustrated that downregulation of PAICS led to a reduction in PKM2, GLUT1, Vimentin, N-cadherin, Zeb1, Snail, and Slug levels, while elevating those of E-cadherin (Fig. 5B, C and Supplementary Fig. 7B, C). Moreover, p-FAK expression levels were significantly reduced following downregulation of PAICS (Fig. 5I and Supplementary Fig. 7I).

Downregulation of PAICS further contributed to reductions in the glycolysis rate, glucose consumption, and lactic acid content (Fig. 5D, E and Supplementary Fig. 7D, E). Moreover, downregulation of PAICS brought about inhibited EMT in breast cancer (Fig. 5F and Supplementary Fig. 7F). In addition, repressed cell migration and invasion abilities were documented following downregulation of PAICS (Fig. 5G, H and Supplementary Fig. 7G, H). Altogether, these findings indicated that downregulation of FAK could inhibit glycolysis, EMT, migration, and invasion of breast cancer cells.

### PAICS reverses the suppressive role of miR-4731-5p in glycolysis, EMT, migration, and invasion of breast cancer cells

**Fig. 6:**
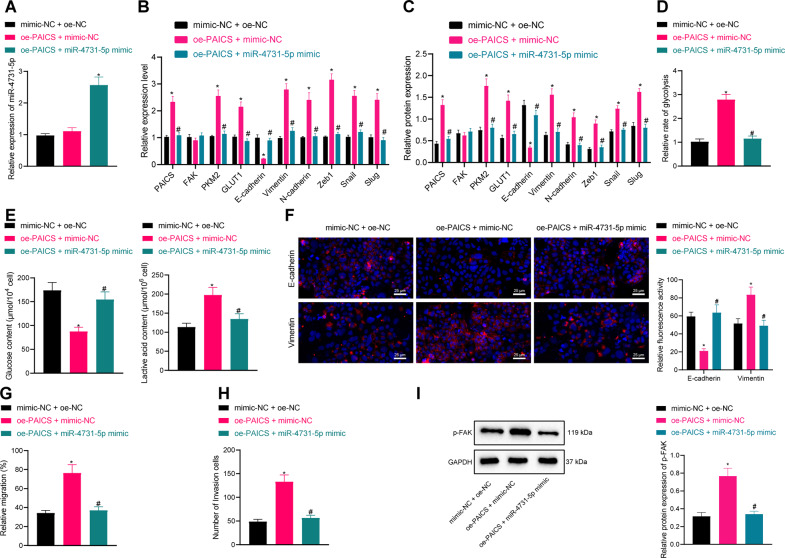
miR-4731-5p inhibits the glycolysis and EMT of MCF-7 cells through regulation of PAICS-mediated phosphorylation of FAK. **A** RT-qPCR was used to detect the expression of miR-4731-5p in breast cancer cells after oe-PAICS and miR-4731-5p mimic treatment. **B** RT-qPCR was used to detect the expression of PAICS, FAK, PKM2, GLUT1, Vimentin, N-cadherin, Zeb1, Snail and Slug and E-Cadherin in cells after oe-PAICS/miR-4731-5p mimic treatment. **C** Western blot analysis was used to detect the expression of PAICS, p-FAK, PKM2, GLUT1, Vimentin, N-cadherin, Zeb1, Snail and Slug and E-Cadherin in cells after oe-PAICS/miR-4731-5p mimic treatment. **D** The effect of oe-PAICS/miR-4731-5p mimic on glycolysis of breast cancer cells. **E** The effect of oe-PAICS/miR-4731-5p mimic on glucose and lactic acid content of breast cancer cells. **F** Immunofluorescence was used to detect the expression of EMT-related markers Vimentin and E-Cadherin, scale bar = 25 μm. **G** Detection of cell migration by scratch test after oe-PAICS/miR-4731-5p mimic treatment. **H** Detection of cell invasion by Transwell assay after oe-PAICS/miR-4731-5p mimic treatment. **I** Western blot analysis was used to detect the expression of p-FAK after oe-PAICS/miR-4731-5p mimic treatment. Measurement data were expressed as mean ± standard deviation. Comparison between two groups was performed through an independent *t* test. Analysis among multiple groups was conducted by one-way ANOVA followed by Tukey’s post hoc test. *p < 0.05 compared with mimic NC + oe-NC treatment. ^#^*p* < 0.05 compared with oe-PAICS + mimic NC treatment.

Furthermore, we validated the underlying mechanism of the miR-4731-5p/PAICS/p-FAK axis in breast cancer. The results of RT-qPCR illustrated that individual oe-PAICS treatment led to increased PAICS expression levels, while further miR-4731-5p mimic increased the miR-4731-5p expression and reduced PAICS levels (Fig. 6A, B and Supplementary Fig. 8A, B). Besides, overexpression of PAICS gave rise to increased PAICS, PKM2, GLUT1, Vimentin, N-cadherin, Zeb1, Snail, and Slug expression levels, and decreased E-cadherin expression, whereas the addition of over-expressed miR-4731-5p reversed the aforementioned trends (Fig. 6B, C and Supplementary Fig. 8B, C). Moreover, there was a significant increase in the expression of p-FAK after individual oe-PAICS treatment, while being reduced following miR-4731-5p mimic treatment (Fig. 6I and Supplementary Fig. 8I).

Subsequent results of glycolysis experimentation illustrated that oe-PAICS treatment brought about a significant increase in the glycolysis rate, glucose consumption, and lactic acid production, while these promotions were inhibited by miR-4731-5p mimic treatment (Fig. 6D, E and Supplementary Fig. 8D, E). Immunofluorescence results further demonstrated that oe-PAICS induced the EMT of tumor cells, while miR-4731-5p mimic countered this effect (Fig. 6F and Supplementary Fig. 8F). Additionally, overexpression of PAICS led to an enhancement in the migration and invasion of breast cancer cells, whereas miR-4731-5p mimic treatment led to the opposing trends (Fig. 6G, H and Supplementary Fig. 8G, H). Altogether, these findings indicated that PAICS could reverse the suppressive role of miR-4731-5p in glycolysis, EMT, migration, and invasion of breast cancer cells.

### miR-4731-5p represses in vivo tumorigenesis and metastasis in breast cancer through PAICS/p-FAK

**Fig. 7:**
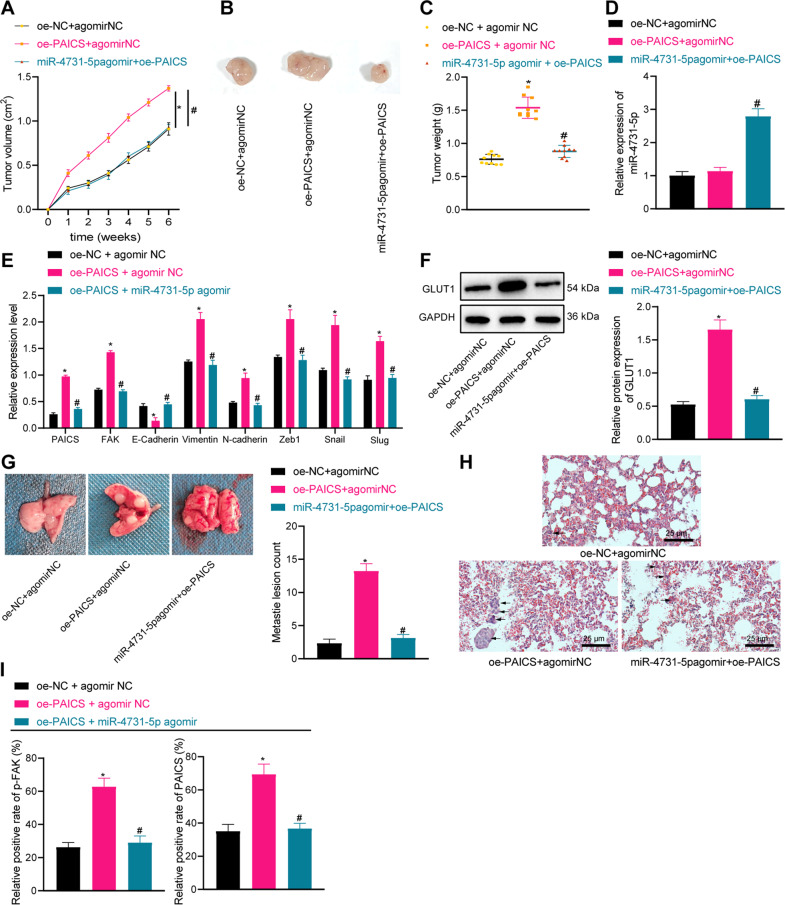
miR-4731-5p inhibits in vivo tumorigenesis and metastasis in breast cancer through PAICS/p-FAK. **A** The volume of tumor in nude mice was statistically analyzed. **B** Representative images of xenograft tumors. **C** Tumor weight in mice after oe-PAICS and miR-4731-5p agomir treatment. **D** RT-qPCR was used to detect the expression of miR-4731-5p in tumors after oe-PAICS and miR-4731-5p agomir treatment. **E** RT-qPCR was used to detect the expression of PAICS, p-FAK, Vimentin, N-cadherin, Zeb1, Snail, Slug, and E-Cadherin. **F** Western blot analysis was used to detect the expression of GLUT1 in tumors. **G** The lung and lung tumor metastases in mouse models were observed (the arrow indicates the number of lung metastases). **H** H&E staining was used to observe the metastasis of lung tissue, scale bar = 25 μm. **I** The expression of p-FAK and PAICS in nude mice was detected by immunohistochemistry. Measurement data were expressed as mean ± standard deviation. Analysis among multiple groups was conducted by one-way ANOVA followed by Tukey’s post hoc test, *n* = 10. * *p* < 0.05 compared with oe-NC + agomir NC treatment. ^#^*p* < 0.05 compared with oe-PAICS + agomir NC treatment.

To further validate the effects of the miR-4731-5p/PAICS/p-FAK axis on breast cancer in vivo, we established a nude mouse model of breast cancer xenograft tumor. There was an increase in tumor volume and weight following PAICS overexpression, while additional treatment with miR-4731-5p agomir reversed these trends (Fig. 7A–C). Moreover, miR-4731-5p expression levels were increased by the injection of miR-4731-5p agomir in the presence of oe-PAICS (Fig. 7D). Meanwhile, overexpression of PAICS augmented the expressions of PAICS, p-FAK, Vimentin, N-cadherin, Zeb1, Snail, and Slug, and reduced that of E-Cadherin in the tumors, whereas additional treatment with miR-4731-5p agomir reversed the aforementioned trends (Fig. 7E). In addition, oe-PAICS treatment gave rise to elevated expressions of glucose metabolism-related protein GLUT1 in tumor tissues, while further miR-4731-5p agomir treatment countered the said effect on GLUT1 expression (Fig. 7F).

Furthermore, we established breast cancer lung metastasis models and counted the metastatic tumor nodules 5 weeks later, and found that oe-PAICS treatment increased lung tumor metastases, whereas further miR-4731-5p agomir treatment decreased lung tumor metastases in nude mice (Fig. 7G). The results of Hematoxylin and Eosin staining further illustrated that lung metastases showed typical adenocarcinoma nests in nude mice after oe-PAICS treatment (Fig. 7H). As evidenced by immunohistochemistry results, overexpression of PAICS gave rise to increased expressions of p-FAK and PAICS, while additional treatment with miR-4731-5p agomir reversed the trends (Fig. 7I). We also documented similar expression trends concerning the expression of p-FAK and PAICS in the breast cancer lung metastasis model as in the nude mouse model of breast cancer xenograft tumor by immunohistochemistry (Supplementary Fig. 9). Altogether, the aforementioned findings indicated that miR-4731-5p could repress in vivo tumorigenesis and metastasis in breast cancer through PAICS/p-FAK.

## Discussion

**Fig. 8:**
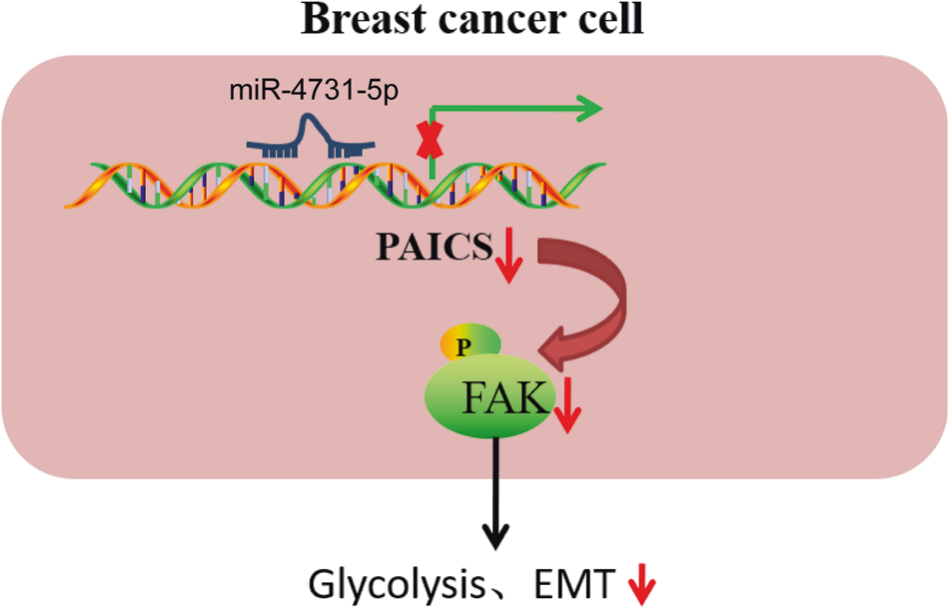
Schematic map of the underlying mechanism concerning the role of miR-4731-5p in breast cancer. miR-4731-5p inhibited the glycolysis and EMT of breast cancer cells via downregulation of p-FAK through reducing PAICS expression.

The current study set out to unravel the function of miR-4731-5p in breast cancer progression, and the obtained findings highlighted the antitumor activity of miR-4731-5p in breast cancer through suppression of glycolysis and reversal of EMT. In addition, we also uncovered a regulatory axis, wherein miR-4731-5p targeted PAICS and consequently inhibited phosphorylation of FAK which impeded glycolysis and blocked the EMT process (Fig. 8).

Initial findings in our study revealed that miR-4731-5p was poorly expressed in breast cancer, whereas its re-expression exerted a diminishing effect on the glycolysis of breast cancer cells and their migration and invasive abilities. Despite the lack of research in regard to the role of miR-4731-5p in breast cancer, the hard-done work of our peers has shed light on the tumor-suppressive role of miRNAs in various malignancies, including stage IV melanoma [7], and glioblastoma [9]. More importantly, downregulation of miR-4731-5p, the focus of our study, was previously detected in oral squamous cell carcinoma, and further correlated with malignant transformation of oral lichen planus as well as malignant characteristics [19].

Further experimentation in our study established that the PAICS oncogene served as a target gene of miR-4731-5p. Moreover, PAICS knockdown led to suppression of glycolysis, EMT, migration, and invasion in breast cancer cells, similar to the effects precipitated by re-expression of miR-4731-5p. In line with our results, Meng et al. previously indicated that downregulation of PAICS could prevent the malignant progression of breast cancer [12]. Furthermore, a number of prior studies have elaborated on the role of PAICS in several cancers. For instance, depletion of PAICS suppressed the proliferative and invasive abilities of pancreatic ductal adenocarcinoma cells [20]. On the other hand, PAICS, activated in LADC tissues, was previously shown to function as a tumor-promoting gene [10]. Meanwhile, loss of PAICS can not only impede the growth of gastric cancer cells but also induces their apoptosis and DNA damage [21]. Further in line with our findings, PAICS is capable of reversing the blockade of EMT triggered by miR-504 [11]. Similarly, our findings validated that miR-4731-5p could reverse the promotive role of PAICS in the malignant progression, including that on glycolysis and EMT induction, which made it plausible to suggest that miR-4731-5p prevented malignant progression of breast cancer via downregulation of PAICS.

Subsequent experimentation in our study unraveled that, PAICS activated FAK by inducing its phosphorylation. FAK, well-established as a potent promoter of breast cancer malignancy [22], is also implicated in various pathways associated with cancer cell growth and glycolysis. For example, activated FAK/Syk/Akt/mTOR and STAT3 pathways are known to underlie Reelin-driven cancer cell growth and metabolic reprogramming, including glycolysis in myeloma cells [23]. Moreover, FAK activation was previously shown to reverse miR-4324-induced EMT inactivation in esophageal squamous cell carcinoma cells [24]. The latter is particularly noteworthy as EMT programs are essential for the facilitation of breast cancer invasion, and micrometastases are also partially-dependent on FAK [25]. FAK also possesses the ability to induce EMT and cisplatin resistance in breast cancer cells [26]. Meanwhile, the FAK enzymatic activity was previously attributed to β3 integrin-induced fibroblast growth factor receptor (FGFR) signaling that physically disrupts the interaction between FGFR1 and E-cadherin, paving the way for metastatic outgrowth [27]. Further, in accordance with our findings, a prior study documented that inactivation of the αβ3 integrin/FAK/PI3K/Akt pathway served as a critical mechanism for effective blockade of EMT by oxymatrine in breast cancer cells [28]. In addition, activated SRC after phosphorylation at Y418 and FAK phosphorylation at Y861 and Y925 of SRC substrate sites, were previously illustrated to be closely associated with the EMT phenotype [29]. Besides, another study established that leptin elevates the expression levels of Twist and β-catenin, as well as the release of invasion markers MMP-2 and MMP-9 in breast cancer cells, leading to enhanced cell invasion in an SRC- and FAK-dependent way [30]. Interestingly, blocking the SRC/EGFR-dependent ITGB4/FAK pathway, which subsequently inactivates the Akt and p38/MAPK pathways contributes to the suppression of migration and invasiveness of breast cancer cells and reversal of EMT process after treatment with Ziyu II [31]. Herein, we unfolded that overexpression of PAICS could promote cell glycolysis and induced EMT by phosphorylating FAK, which could potentially serve as the mechanism contributing to breast cancer progression due to loss of miR-4731-5p.

Collectively, our findings highlighted the tumor-suppressive and anti-metastatic roles of miR-4731-5p in breast cancer. In addition, we proposed a mechanism wherein miR-4731-5p represses PAICS-dependent phosphorylation of FAK, whereby suppressing the glycolysis of breast cancer cells and reversing EMT process. Although we successfully substantiated the tumor-suppressive and anti-metastatic functions of miR-4731-5p agomir in nude mouse models, it would be prudent to carry out further clinical trials with large sample sizes before therapeutic use.

## Methods

### Ethics statement

All research protocols were approved by Chongqing University Central Hospital and in accordance with the *Declaration of Helsinki*. Signed informed consents were obtained from all participants prior to specimen collection. Animal experiment protocols were sanctioned by the local Animal Ethics Committee of Chongqing Medical University. Extensive efforts were undertaken to minimize both the number and suffering of the experimental animals.

### Bioinformatics analysis

First, breast cancer-related miRNA microarray data set GSE57897 (comprising of 31 normal samples and 422 breast cancer samples) and mRNA microarray data set GSE3744 (comprising of seven normal samples and 40 breast cancer samples) were retrieved from the Gene Expression Omnibus (GEO) database. Differentially expressed genes (|logFC | > 0.8, adj.*p*.val < 0.05) were identified using the R language “limma” package, with normal samples as controls. The different *p* values were corrected with the false discovery rate method. The false-positive rate was decreased by adjusted *p* values using the Benjamini and Hochberg false discovery rate method by default. Additionally, the target genes of miR-4731-5p were obtained from the starBase and TargetScan databases. Furthermore, the differentially expressed genes in the TCGA and GTEx databases were downloaded using the GEPIA server. Thereafter, an interaction analysis was performed on the candidate target genes with the interaction scores obtained with the help of the GeneMANIA database.

### Sample collection

A total of 50 patients who underwent breast tumor resection at the Chongqing University Central Hospital from January 2017 to December 2020, were enrolled in the current study to obtain breast cancer tissues and adjacent normal tissues. The included patients had an age range of 37–72 years, with a mean calculated age of (57.68 ± 7.30) years. None of the included patients had received antitumor therapy prior to specimen collection and were confirmed as breast cancer by means of postoperative pathological biopsy.

### Cell culture and transfection

Breast cancer cell lines (namely, MDA-MB-436, MDA-MB-453, MCF-7, and MDA-MB-231) were all purchased from ATCC (Manassas, VA). The obtained cells were cultured in RPMI-1640 (Gibco, USA) containing 10% fetal bovine serum (FBS, Gibco), 100 μg/mL streptomycin, and 100 U/mL penicillin, and placed in a humidified incubator at 37°C with 5% CO_2_ in air (Thermo Fisher, Waltham, MA). Cells at the logarithmic phase were trypsinized and inoculated in a six-well plate, at a density of 1 × 10^5^ cells per well. Following a 24-h period of regular culture, the cells were transfected upon reaching 75% confluence. The concentration of NC, miRNA mimic, or miRNA inhibitor was set as 50 nM (RiboBio Biotechnology Co., Ltd., Guangzhou, China). Transfection was carried out using the Lipofectamine 3000 (L3000001, Thermo Fisher) reagent.

### RT-qPCR

Total RNA content was extracted from the aforementioned cells using the TRIzol reagent (15596018, Solarbio, Beijing, China). The primers used in the current study were synthesized by Sangon (Shanghai, China) (Supplementary Table 1). For miRNA detection, the miRNA one-step reverse transcription kits (D1801, Harbin Xinhai Genetic Testing Co., Ltd., Harbin, China) were utilized with miRNA fluorescence quantitative PCR (AQ101-02, Quanshijin, Beijing). Meanwhile, for mRNA detection, RNA reverse transcription kits (KR116, Tiangen Biotechnology Co., Ltd., Beijing, China) were adopted with a fluorescent quantitative PCR instrument Applied Biosystems ViiA™ 7 (LifeTechnologies, Inc., Applied Biosystems, Foster City, CA). Transcriptional levels of the genes were calculated using the 2^−^^ΔΔCt^ quantification method, with β-actin and U6 serving as internal references. The absolute copy number of each miRNA was determined with a standard curve. To this end, miRNA oligonucleotides (IDT, Iowa, Coralville) were utilized to produce standard curves at various concentrations (1 × 10^1–8^ molecules/reaction). The sequence of mature miRNA was CCUAAUUUGAACACCUUCGGUA.

### Western blot analysis

Western blot analysis was carried out with following primary rabbit antibodies: PKM2 (ab137852, 1:1000), GLUT1 (ab115730, 1:1000), PAICS (ab229531, 1:1000), FAK (ab40794, 1:1000), p-FAK (ab81298, 1:1000), Snail (ab216347, 1:1000), Slug (ab27568, 1:1000), N-cadherin (ab76011, 1:1000), Zeb1 (ab203829, 1:1000), E-cadherin (ab40772, 1:1000), Vimentin (ab92547, 1:1000), and glyceraldehyde-3-phosphate dehydrogenase (GAPDH; ab9485, 1:1000), and the secondary antibody horseradish peroxidase-labeled goat anti-rabbit immunoglobulin (ab205718, 1: 20000). The protein quantitative analysis was performed with the gray value of each protein and the gray ratio of the internal reference GAPDH using the ImageJ software. All the aforementioned antibodies were purchased from Abcam (Cambridge, UK).

### Glycolysis assay

The degree of glycolysis in breast cancer cells under different conditions was detected by means of a glycolysis assay (ab197244, Abcam). Meanwhile, the degree of glucose consumption in breast cancer cells was measured using the micro method (BC2505, Solarbio). Briefly, the collected cells were centrifuged with the supernatant removed. According to the ratio of 500:1–1000:1 of the number of cells (10,000 cells): volume of distilled water (mL), the cells were broken by ultra-sonification (ice bath, power 20% or 200 W, ultrasonic 3 s, 10 s interval, repeated for 30 times) and then water-bathed at 95°C for 10 min (cover tightly to prevent water loss). After being allowed to cool down, the cells were centrifuged at 8000 × *g* at 25°C for 10 min, with the supernatant collected for later use. A spectrophotometer/plate reader was subsequently warmed up for >30 min, with the wavelength adjusted to 505 nm for detection purposes.

Additionally, the micro method was applied another time for detection of the degree of lactic acid production (BC2235, Solarbio), and the steps were the same as those for glucose detection. The spectrophotometer/plate reader was warmed up for more than 30 min, with the wavelength adjusted to 570 nm for detection purposes.

### Scratch test

After detachment, cells were seeded in a 24-well plate, and upon reaching 90% cell confluence, scratches were made using a sterile pipette tip. After a phosphate-buffered saline (PBS) rinse, the corresponding treatment was performed. Followed a 48-h treatment period, images were obtained. The ImageJ software was adopted to calculate the scratch area.

### Transwell assay

Breast cancer cells were cultured in a serum-free medium for 12 h, harvested, and resuspended in a serum-free medium (1 × 10^5^/ml). The lower chamber was added with 10% FBS, and 100 μL of cell suspension was added to the Transwell chamber (PIEP12R48, Millipore, Darmstadt, Germany) coated with Matrigel (356231, Corning BioCoat, Bedford). Following a 24-h incubation period at 37°C, the cells that did not invade the cell surface were gently removed with cotton swabs. The remaining cells were then fixed with 100% methanol and stained with 20% crystal violet (Sigma). The stained invaded cells were counted manually under five randomly selected areas using an inverted optical microscope (CarlZeiss, Jena, Germany), and each experiment was repeated three times to obtain the mean value. The procedure of the migration experiment was the same without Matrigel.

### Dual-luciferase reporter gene assay

The TargetScan webserver was adopted to predict the targeted binding site. The artificially-synthesized WT and MUT of the PAICS 3′-UTR region were inserted into the luciferase reporter vector pmiR-RB-Report™ (Cat. No.: E1330, Promega, Madison, WI). Subsequently, the 3′-UTR luciferase vector (150 ng) and miR-4731-5p mimic (50 nmol/L) or NC were co-transfected into breast cancer cells using the Lipo 3000 reagent (Invitrogen, Carlsbad, CA). The luciferase activity was detected using luciferase detection kits (Promega) and a Glomax20/20 luminometer (Promega).

### Immunofluorescence

Following cell-processing, the medium was removed at the corresponding time points, and the cells were rinsed with PBS thrice, fixed with 4% paraformaldehyde for 15 min, and then stored at 4°C for short-term storage. Subsequently, the cells were rinsed thrice with a PBS solution containing 0.1% Tween-20, PBS solution containing 0.25% Triton X-100 (PBST) at room temperature for 10 min, and PBST solution containing 1% BSA + 22.52 mg/ml Glycine + 0.1% Tween-20 for 30 min at room temperature. Next, the cells were incubated with the following corresponding primary antibodies: rabbit anti-E-cadherin (ab40772, 1:100, Abcam) and rabbit anti-Vimentin (ab92547, 1:100, Abcam) at 4°C overnight. The following day, the cells were rinsed thrice with PBST solution and incubated with the corresponding fluorescent secondary antibody at room temperature for 1 h. Afterward, the cells were stained with 1 µg/mL DAPI at room temperature for 5 min, rinsed thrice with PBST solution, followed by observation and photography with a fluorescence microscope.

### Immunohistochemistry

Paraffin-embedded sections were fixed with formalin and sectioned (thickness of 4 μm), dewaxed in xylene, and rehydrated. Endogenous peroxidase activity was quenched with the addition of 3% hydrogen peroxide dissolved in methanol for 10 min, and the sections were subjected to antigen repair for 15 min. Next, the sections were blocked for 20 min with normal rabbit serum and incubated with anti-p-FAK (Abcam) and anti-PAICS (Abcam) overnight at 4°C. The following day, the sections were incubated with the secondary antibody for 30 min, and stained with streptavidin peroxidase detection system, counterstained with hematoxylin with diaminobenzidine as a chromogenic agent. PBS was adopted as the NC.

### Xenograft tumor in nude mice

A total of 60 healthy female nude mice (aged 4–6 weeks old, procured from Shanghai SLAC Laboratory Animal Co., Ltd) were raised in an SPF animal laboratory in single cages under controlled conditions (laboratory humidity: 60–65%; temperature: 22–25°C). All mice had ad libitum access to food and water and were raised under 12 h light and dark cycles. The experiment was performed after 1 week of acclimation and the health of the nude mice was observed prior to the experiment.

MDA-MB-231 cells were transduced with 50 nM of agomir NC, oe-NC, oe-PAICS, or miR-4731-5p agomir alone or in combination to establish stably transduced cells. agomir NC, and miR-4731-5p agomir were all purchased from Sangon. Subsequently, 10 nude mice were selected from each group and injected with MDA-MB-231 cell suspension (2 × 10^6^ cells/50 μL, 1 × 10^7^ cells/mL), which was prepared using MDA-MB-231 cells at the logarithmic phase of growth, into the skin under the left armpit to induce xenograft tumors. Mice were euthanized by cervical dislocation 6 weeks later, followed by tumor growth observation. Tumor weight was detected using a balance.

MDA-MB-231 (density of 1 × 10^5^ cells/50 µL) cells were injected into nude mice (*n* = 10/group) via a tail vein injection to establish breast cancer lung, metastasis models. Mice were euthanized by cervical dislocation 5 weeks later, and the metastatic tumor nodules were collected.

### Statistical analysis

Statistical analyses were processed using the SPSS21.0 software (IBM Corp, Armonk, NY, USA). Measurement data were presented as mean ± standard deviation. Comparisons between two groups were performed through independent *t* tests. Analysis among multiple groups was conducted by one-way analysis of variance, followed by Tukey’s post hoc test. A value of *p* < 0.05 was regarded statistically significant. All experiments were repeated at least three times independently.

## Supplementary information

full and uncropped western blots

supplementary files

## Data Availability

The data that support the findings of this study are available on request from the corresponding author upon reasonable request.
