# Supplementary material for: Tumor suppressive role of microRNA-4731-5p in breast cancer through reduction of PAICS-induced FAK phosphorylation
Source: Cell Death Discov. 2022 Apr 4;8:154. doi: 10.1038/s41420-022-00938-1 (PMC8980087; doi:10.1038/s41420-022-00938-1)
Supplement: Supplementary file 2 — supplementary files [file 41420_2022_938_MOESM2_ESM.docx]

**Table S1** Primer sequences for RT-qPCR

|  | Sequences (5'-3') |
| --- | --- |
| *miR-4731-5p* | F: GGGGGCCACATGAGT |
|  | R: GGTCCAGTTTTTTTTTTTTTTTCACA |
| *Snail* (human) | F: TGCCCTCAAGATGCACATCCGA |
|  | R: GGGACAGGAGAAGGGCTTCTC |
| *Slug* (human) | F: ACGCCTCCAAAAAGCCAAAC |
|  | R: ACTCACTCGCCCCAAAGATG |
| *Zeb1* (human) | F: GATGATGAATGCGAGTCAGATGC |
|  | R: ACAGCAGTGTCTTGTTGTTGT |
| *N-cadherin* (human) | F: TTTGATGGAGGTCTCCTAACACC |
|  | R: ACGTTTAACACGTTGGAAATGTG |
| *PKM2* (human) | F: ATGGCTGACACATTCCTGGAGC |
|  | R: CCTTCAACGTCTCCACTGATCG |
| *GLUT1* (human) | F: GGCCAAGAGTGTGCTAAAGAA |
|  | R: ACAGCGTTGATGCCAGACAG |
| *PAICS* (human) | F: ACCACCTGGAAGGAAAAGCTGC |
|  | R: CGGTGCAATGAAAGCTGTCTCC |
| *FAK* (human) | F: GCTTACCTTGACCCCAACTTG |
|  | R: ACGTTCCATACCAGTACCCAG |
| *E-cadherin* (human) | F: CGAGAGCTACACGTTCACGG |
|  | R: GGGTGTCGAGGGAAAAATAGG |
| *Vimentin* (human) | F: GACGCCATCAACACCGAGTT |
|  | R: CTTTGTCGTTGGTTAGCTGGT |
| *β-actin* (human) | F: CATGTACGTTGCTATCCAGGC |
|  | R: CTCCTTAATGTCACGCACGAT |
| *U6* (human) | F: AACGCTTCACGAATTTGCGT |
|  | R: TTCACGAATTTGCGTGTCAT |

Note: RT-qPCR, reverse transcription quantitative polymerase chain reaction; F, forward; R, reverse.

**Table S2 Information of related intersection genes**

| Symbol | Score | Functions | Links |  |  |  |  |  |  |  |
| --- | --- | --- | --- | --- | --- | --- | --- | --- | --- | --- |
| SBK1 | 0.804144 |  | http://www.ncbi.nlm.nih.gov/sites/entrez?db=gene&cmd=search&term=388228 | | | | | | | |
| CTXN1 | 0.799702 |  | http://www.ncbi.nlm.nih.gov/sites/entrez?db=gene&cmd=search&term=404217 | | | | | | | |
| SAPCD2 | 0.774058 |  | http://www.ncbi.nlm.nih.gov/sites/entrez?db=gene&cmd=search&term=89958 | | | | | | | |
| VANGL1 | 0.722965 |  | http://www.ncbi.nlm.nih.gov/sites/entrez?db=gene&cmd=search&term=81839 | | | | | | | |
| PLEK2 | 0.69239 |  | http://www.ncbi.nlm.nih.gov/sites/entrez?db=gene&cmd=search&term=26499 | | | | | | | |
| SPC24 | 0.662342 | chromosome, centromeric region, kinetochore, protein-DNA complex | http://www.ncbi.nlm.nih.gov/sites/entrez?db=gene&cmd=search&term=147841 | | | | | | | |
| PRR11 | 0.661073 |  | http://www.ncbi.nlm.nih.gov/sites/entrez?db=gene&cmd=search&term=55771 | | | | | | | |
| PAQR4 | 0.658791 |  | http://www.ncbi.nlm.nih.gov/sites/entrez?db=gene&cmd=search&term=124222 | | | | | | | |
| PAICS | 0.646098 |  | http://www.ncbi.nlm.nih.gov/sites/entrez?db=gene&cmd=search&term=10606 | | | | | | | |
| PITX1 | 0.645185 |  | http://www.ncbi.nlm.nih.gov/sites/entrez?db=gene&cmd=search&term=5307 | | | | | | | |
| CDCA3 | 0.640045 |  | http://www.ncbi.nlm.nih.gov/sites/entrez?db=gene&cmd=search&term=83461 | | | | | | | |
| HMGA1 | 0.63848 | DNA replication, DNA-dependent DNA replication, protein-DNA complex subunit organization | http://www.ncbi.nlm.nih.gov/sites/entrez?db=gene&cmd=search&term=3159 | | | | | | | |
| CDT1 | 0.605191 | cell cycle checkpoint, DNA replication, DNA-dependent DNA replication, G1/S transition of mitotic cell cycle | http://www.ncbi.nlm.nih.gov/sites/entrez?db=gene&cmd=search&term=81620 | | | | | | | |
| SLC7A5 | 0.597214 |  | http://www.ncbi.nlm.nih.gov/sites/entrez?db=gene&cmd=search&term=8140 | | | | | | | |
| MFAP2 | 0.581874 |  | http://www.ncbi.nlm.nih.gov/sites/entrez?db=gene&cmd=search&term=4237 | | | | | | | |
| MCM4 | 0.579813 | ATP-dependent DNA helicase activity, ATP-dependent helicase activity, DNA helicase activity, DNA replication, DNA strand elongation, DNA strand elongation involved in DNA replication, DNA-dependent DNA replication, G1/S transition of mitotic cell cycle, MCM complex, purine NTP-dependent helicase activity | http://www.ncbi.nlm.nih.gov/sites/entrez?db=gene&cmd=search&term=4173 | | | | | | | |
| KIF11 | 0.573788 | microtubule, microtubule-based movement, mitosis, mitotic spindle organization, nuclear division, organelle fission, spindle, spindle microtubule | http://www.ncbi.nlm.nih.gov/sites/entrez?db=gene&cmd=search&term=3832 | | | | | | | |
| FOXM1 | 0.529496 |  | http://www.ncbi.nlm.nih.gov/sites/entrez?db=gene&cmd=search&term=2305 | | | | | | | |
| ASF1B | 0.497563 |  | http://www.ncbi.nlm.nih.gov/sites/entrez?db=gene&cmd=search&term=55723 | | | | | | | |
| CHAF1A | 0.02318 | protein-DNA complex subunit organization | http://www.ncbi.nlm.nih.gov/sites/entrez?db=gene&cmd=search&term=10036 | | | | | | | |
| MCM7 | 0.020916 | ATP-dependent DNA helicase activity, ATP-dependent helicase activity, DNA helicase activity, DNA replication, DNA strand elongation, DNA strand elongation involved in DNA replication, DNA-dependent DNA replication, G1/S transition of mitotic cell cycle, MCM complex, purine NTP-dependent helicase activity | http://www.ncbi.nlm.nih.gov/sites/entrez?db=gene&cmd=search&term=4176 | | | | | | | |
| MCM2 | 0.020286 | DNA replication, DNA strand elongation, DNA strand elongation involved in DNA replication, DNA-dependent DNA replication, G1/S transition of mitotic cell cycle, MCM complex | http://www.ncbi.nlm.nih.gov/sites/entrez?db=gene&cmd=search&term=4171 | | | | | | | |
| HIRA | 0.020081 |  | http://www.ncbi.nlm.nih.gov/sites/entrez?db=gene&cmd=search&term=7290 | | | | | | | |
| MCM5 | 0.01941 | DNA replication, DNA strand elongation, DNA strand elongation involved in DNA replication, DNA-dependent DNA replication, G1/S transition of mitotic cell cycle, MCM complex | http://www.ncbi.nlm.nih.gov/sites/entrez?db=gene&cmd=search&term=4174 | | | | | | | |
| MCM6 | 0.018661 | ATP-dependent DNA helicase activity, ATP-dependent helicase activity, DNA helicase activity, DNA replication, DNA strand elongation, DNA strand elongation involved in DNA replication, DNA-dependent DNA replication, G1/S transition of mitotic cell cycle, MCM complex, purine NTP-dependent helicase activity | http://www.ncbi.nlm.nih.gov/sites/entrez?db=gene&cmd=search&term=4175 | | | | | | | |
| CHAF1B | 0.018566 | protein-DNA complex subunit organization | http://www.ncbi.nlm.nih.gov/sites/entrez?db=gene&cmd=search&term=8208 | | | | | | | |
| MCM3 | 0.016743 | DNA replication, DNA strand elongation, DNA strand elongation involved in DNA replication, DNA-dependent DNA replication, G1/S transition of mitotic cell cycle, MCM complex, protein-DNA complex | http://www.ncbi.nlm.nih.gov/sites/entrez?db=gene&cmd=search&term=4172 | | | | | | | |
| GTSE1 | 0.012472 | cell cycle checkpoint, cell cycle phase, G1/S transition of mitotic cell cycle, microtubule | http://www.ncbi.nlm.nih.gov/sites/entrez?db=gene&cmd=search&term=51512 | | | | | | | |
| KIFC1 | 0.010103 | chromosome segregation, microtubule-based movement, mitosis, mitotic sister chromatid segregation, nuclear division, organelle fission, sister chromatid segregation | http://www.ncbi.nlm.nih.gov/sites/entrez?db=gene&cmd=search&term=3833 | | | | | | | |
| CDCA8 | 0.009913 | cell cycle phase, chromosome, centromeric region, midbody, protein-DNA complex | http://www.ncbi.nlm.nih.gov/sites/entrez?db=gene&cmd=search&term=55143 | | | | | | | |
| MKI67 | 0.009906 |  | http://www.ncbi.nlm.nih.gov/sites/entrez?db=gene&cmd=search&term=4288 | | | | | | | |
| TROAP | 0.009799 |  | http://www.ncbi.nlm.nih.gov/sites/entrez?db=gene&cmd=search&term=10024 | | | | | | | |
| BIRC5 | 0.009786 | cell cycle checkpoint, chromosome localization, chromosome, centromeric region, condensed chromosome, condensed chromosome kinetochore, condensed chromosome, centromeric region, establishment of chromosome localization, kinetochore, microtubule, midbody, mitosis, nuclear division, organelle fission, protein-DNA complex, spindle, spindle checkpoint, spindle microtubule | http://www.ncbi.nlm.nih.gov/sites/entrez?db=gene&cmd=search&term=332 | | | | | | | |
| PLK1 | 0.009667 | cell cycle checkpoint, chromosome segregation, chromosome, centromeric region, condensed chromosome, condensed chromosome kinetochore, condensed chromosome outer kinetochore, condensed chromosome, centromeric region, kinetochore, microtubule, midbody, mitosis, mitotic sister chromatid segregation, nuclear division, organelle fission, protein-DNA complex, sister chromatid segregation, spindle, spindle checkpoint, spindle microtubule | http://www.ncbi.nlm.nih.gov/sites/entrez?db=gene&cmd=search&term=5347 | | | | | | | |
| KIF23 | 0.009638 | microtubule-based movement, midbody, mitosis, mitotic spindle organization, nuclear division, organelle fission, spindle | http://www.ncbi.nlm.nih.gov/sites/entrez?db=gene&cmd=search&term=9493 | | | | | | | |
| HMMR | 0.009597 |  | http://www.ncbi.nlm.nih.gov/sites/entrez?db=gene&cmd=search&term=3161 | | | | | | | |
| GINS2 | 0.009338 | DNA replication, DNA strand elongation, DNA strand elongation involved in DNA replication, DNA-dependent DNA replication | http://www.ncbi.nlm.nih.gov/sites/entrez?db=gene&cmd=search&term=51659 | | | | | | | |
| CENPE | 0.009297 | cell cycle phase, chromosome localization, chromosome segregation, chromosome, centromeric region, condensed chromosome, condensed chromosome kinetochore, condensed chromosome outer kinetochore, condensed chromosome, centromeric region, establishment of chromosome localization, kinetochore, microtubule, microtubule-based movement, mitosis, mitotic sister chromatid segregation, nuclear division, organelle fission, protein-DNA complex, protein-DNA complex subunit organization, sister chromatid segregation | http://www.ncbi.nlm.nih.gov/sites/entrez?db=gene&cmd=search&term=1062 | | | | | | | |
| CCNB1 | 0.009268 | cell cycle checkpoint, chromosome localization, chromosome segregation, chromosome, centromeric region, condensed chromosome, condensed chromosome kinetochore, condensed chromosome outer kinetochore, condensed chromosome, centromeric region, establishment of chromosome localization, G1/S transition of mitotic cell cycle, kinetochore, mitosis, mitotic sister chromatid segregation, mitotic spindle organization, nuclear division, organelle fission, protein-DNA complex, sister chromatid segregation, spindle, spindle checkpoint | http://www.ncbi.nlm.nih.gov/sites/entrez?db=gene&cmd=search&term=891 | | | | | | | |
|  |  |  |  |  |  |  |  |  |  |  |

**
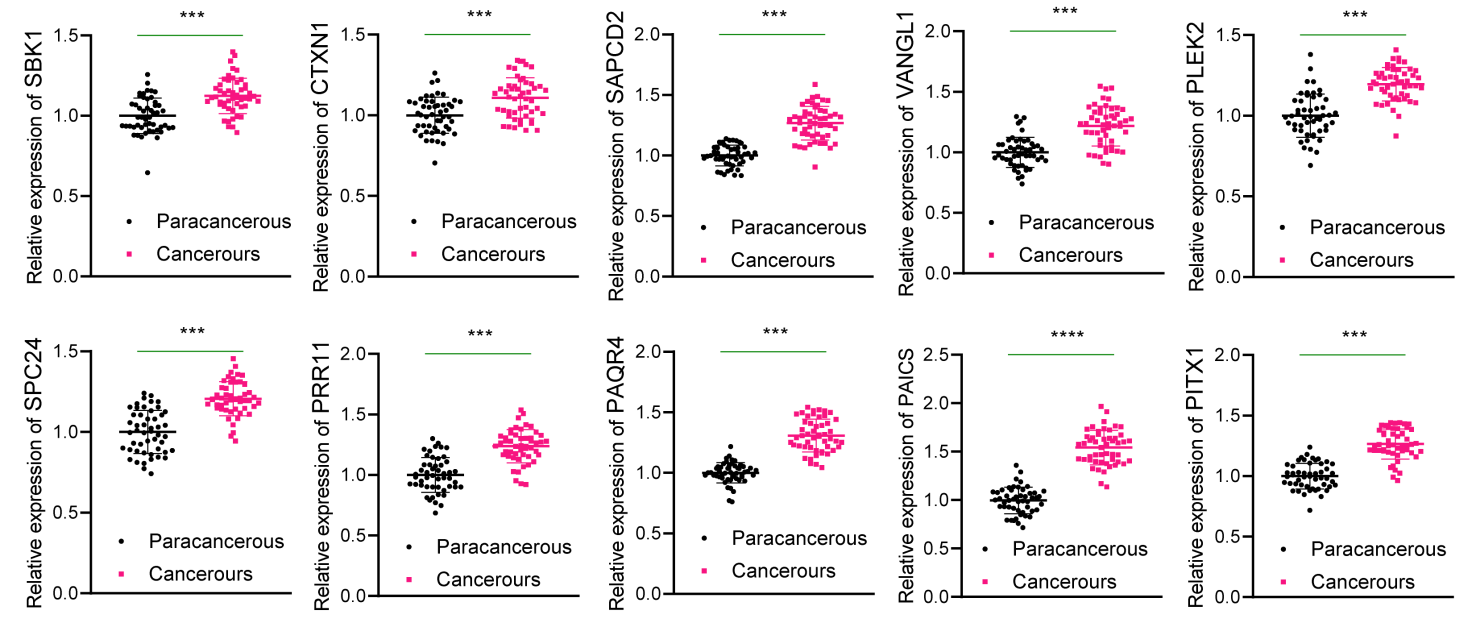
**

**Supplementary Fig. 1** RT-qPCR measurement of SBK1, CTXN1, SAPCD2, VANGL1, PLEK2, SPC24, PRR11, PAQR4, PAICS, and PITX1 expression in clinical samples (n = 50).

**
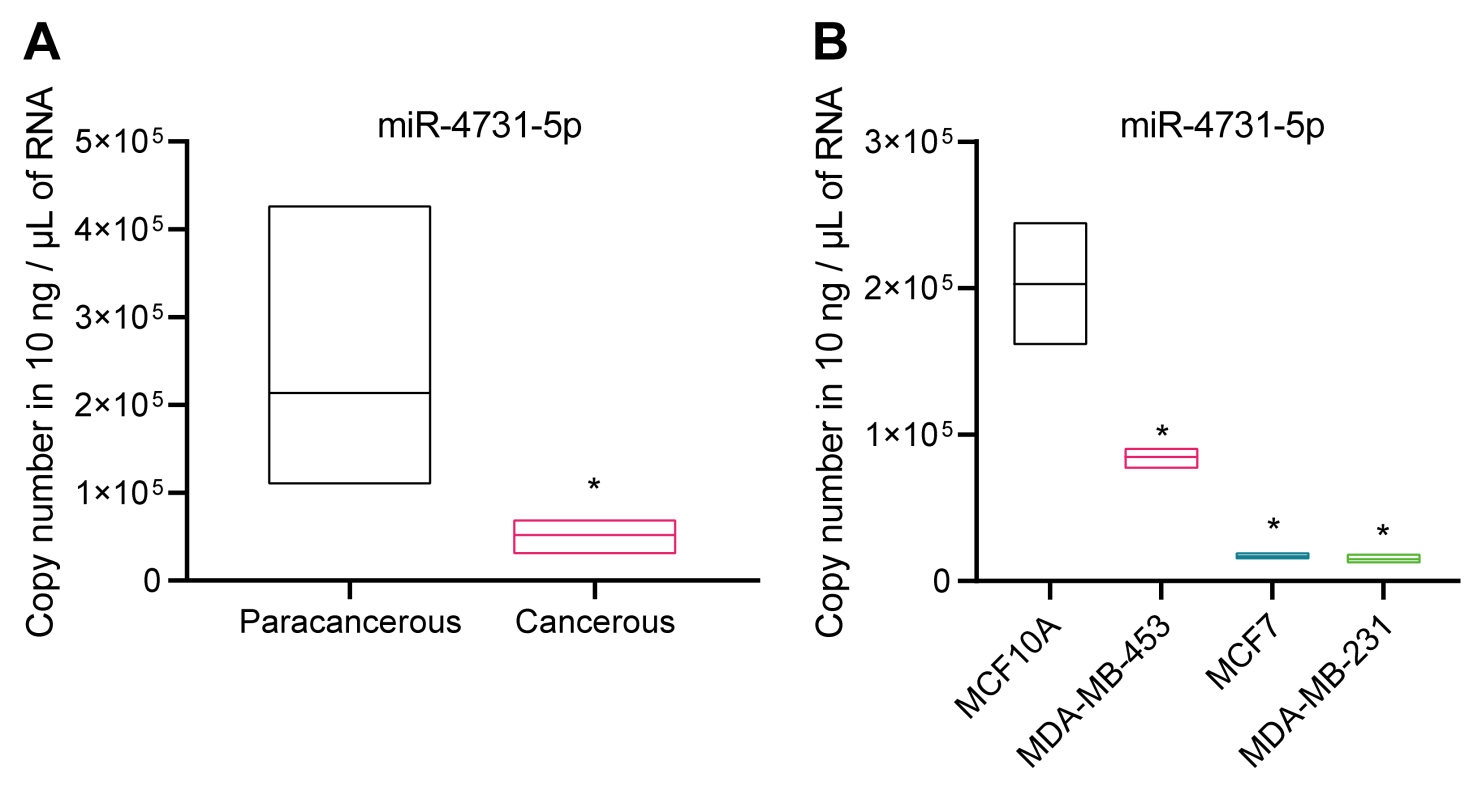
**

**Supplementary Fig. 2** Absolute quantification of miR-4731-5p in clinical samples and cells. A, RT-qPCR was used to detect the expression of miR-4731-5p in clinical samples (n = 50); B, RT-qPCR was used to detect the expression of miR-4731-5p in breast cancer cells (MDA-MB-453, MCF-7, and MDA-MB-231) and breast epithelial cells.

**
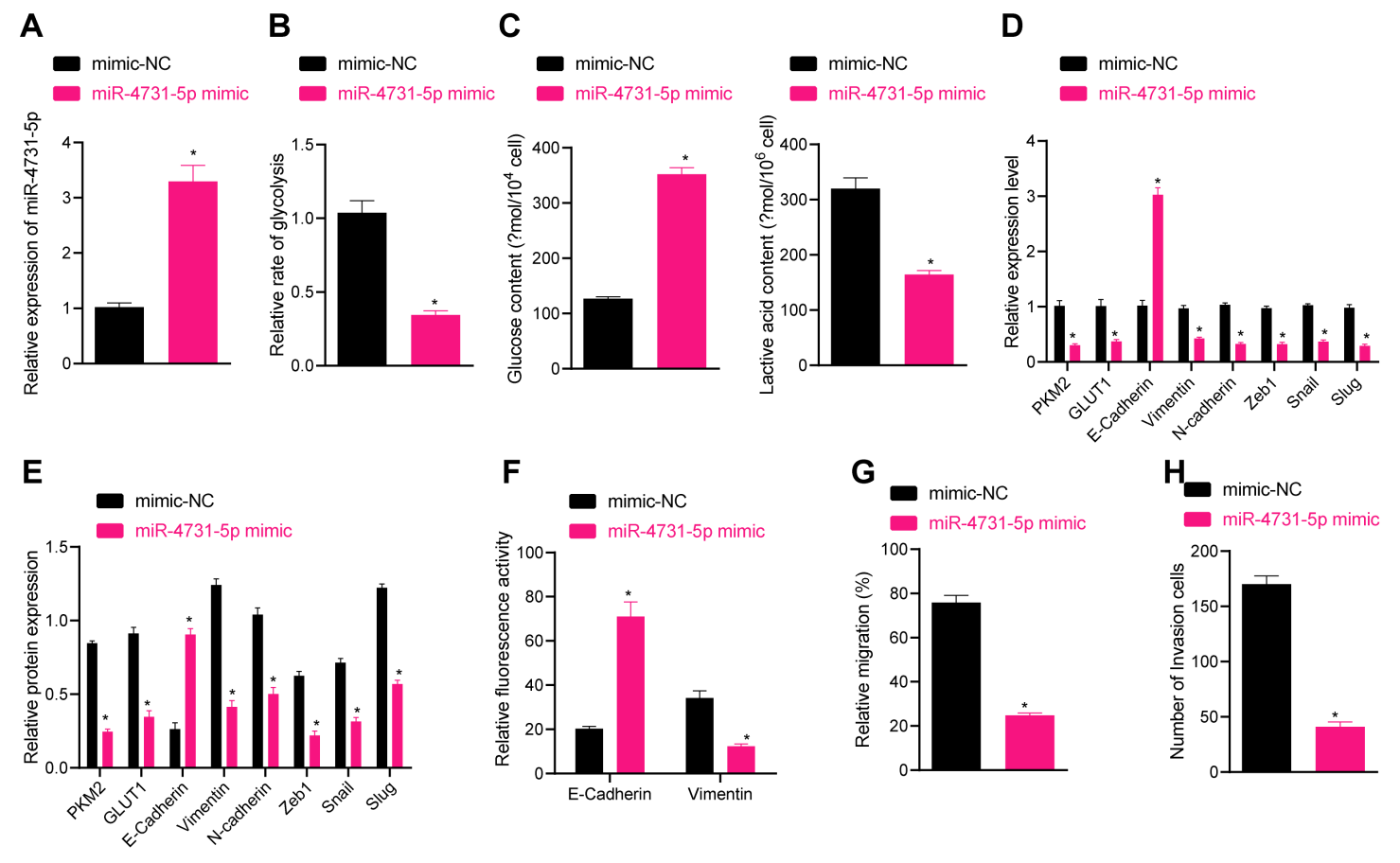
**

**Supplementary Fig. 3** miR-4731-5p suppresses the glycolysis, EMT and migration and invasion of MDA-MB-231 cells. A, RT-qPCR was used to detect the expression of miR-4731-5p after miR-4731-5p mimic treatment; B, The effect of miR-4731-5p on glycolysis of breast cancer cells; C, The effect of miR-4731-5p on glucose and lactic acid content of breast cancer cells; D, RT-qPCR was used to detect the expression of glycolysis-related genes PKM2, GLUT1 and EMT-related markers Vimentin and E-Cadherin; E, Western blot analysis was used to detect the expression of glycolysis-related genes PKM2, GLUT1 and EMT-related markers Vimentin and E-Cadherin. F, Immunofluorescence was used to detect the expression of EMT-related markers Vimentin and E-Cadherin; G, Detection of cell migration by scratch test after miR-4731-5p mimic treatment; H, Detection of cell invasion by Transwell assay after miR-4731-5p mimic treatment; Measurement data were expressed as mean ± standard deviation. Comparison between two groups was performed through independent *t*-test. Analysis among multiple groups was conducted by one-way ANOVA followed by Tukey’s post hoc test. * *p* < 0.05 compared with mimic NC treatment.

**
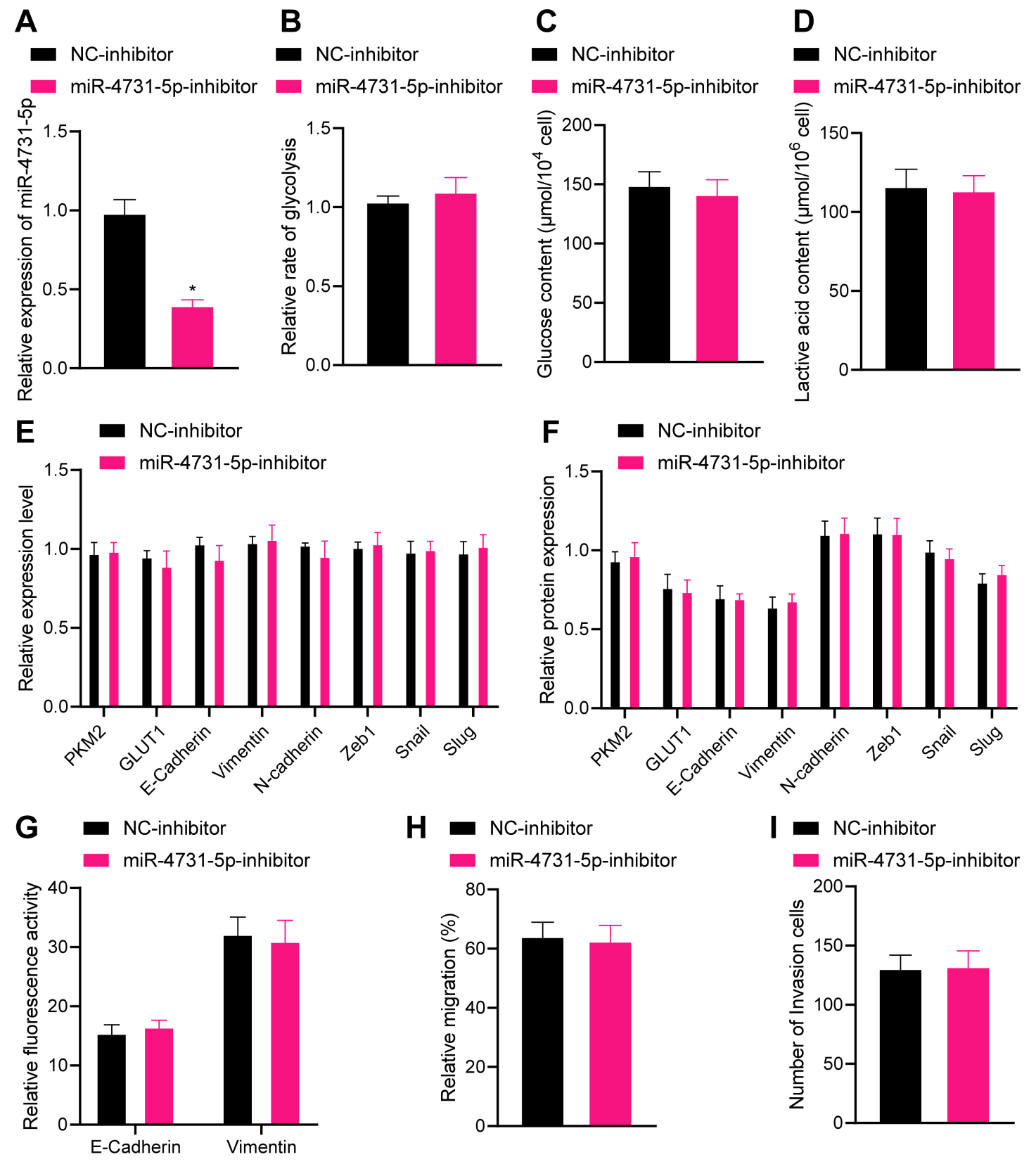
**

**Supplementary Fig. 4** The effect of miR-4731-5p inhibition on the glycolysis and EMT of normal breast cell line MCF10A cells. A, miR-4731-5p expression was determined using RT-qPCR in response to NC inhibitor or miR-4731-5p inhibitor; B, The effect of miR-4731-5p on glycolysis of MCF10A cells; C, The effect of miR-4731-5p on glucose of MCF10A cells; D, The effect of miR-4731-5p on lactic acid content of MCF10A cells; E, RT-qPCR was used to detect the expression of glycolysis-related genes PKM2, GLUT1 and EMT-related markers Vimentin and E-Cadherin in MCF10A cells; F, Western blot analysis was used to detect the expression of glycolysis-related genes PKM2, GLUT1 and EMT-related markers Vimentin and E-Cadherin in MCF10A cells. G, Immunofluorescence was used to detect the expression of EMT-related markers Vimentin and E-Cadherin in MCF10A cells; H, Detection of cell migration by scratch test after miR-4731-5p inhibitor treatment in MCF10A cells; I, Detection of cell invasion by Transwell assay after miR-4731-5p inhibitor treatment in MCF10A cells; Measurement data were expressed as mean ± standard deviation. Comparison between two groups was performed through independent *t*-test. Analysis among multiple groups was conducted by one-way ANOVA followed by Tukey’s post hoc test. * *p* < 0.05 compared with mimic NC treatment.

**
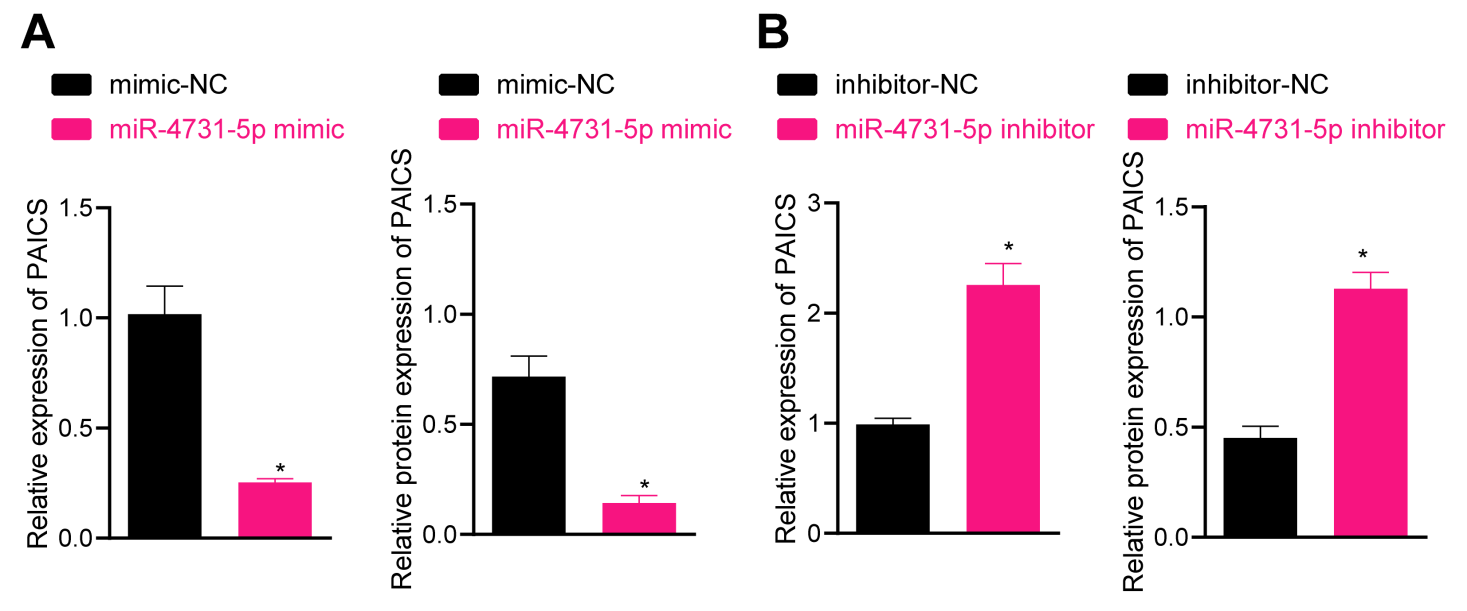
**

**Supplementary Fig. 5** PAICS is the potential target of miR-4731-5p in MDA-MB-231 cells. A, RT-qPCR was used to detect the expression of PAICS after miR-4731-5p mimic treatment; B, Western blot analysis was used to detect the expression of PAICS after miR-4731-5p inhibitor treatment; Measurement data were expressed as mean ± standard deviation. Comparison between two groups was performed through independent *t*-test. * *p* < 0.05 compared with mimic NC or inhibitor-NC treatment.

**
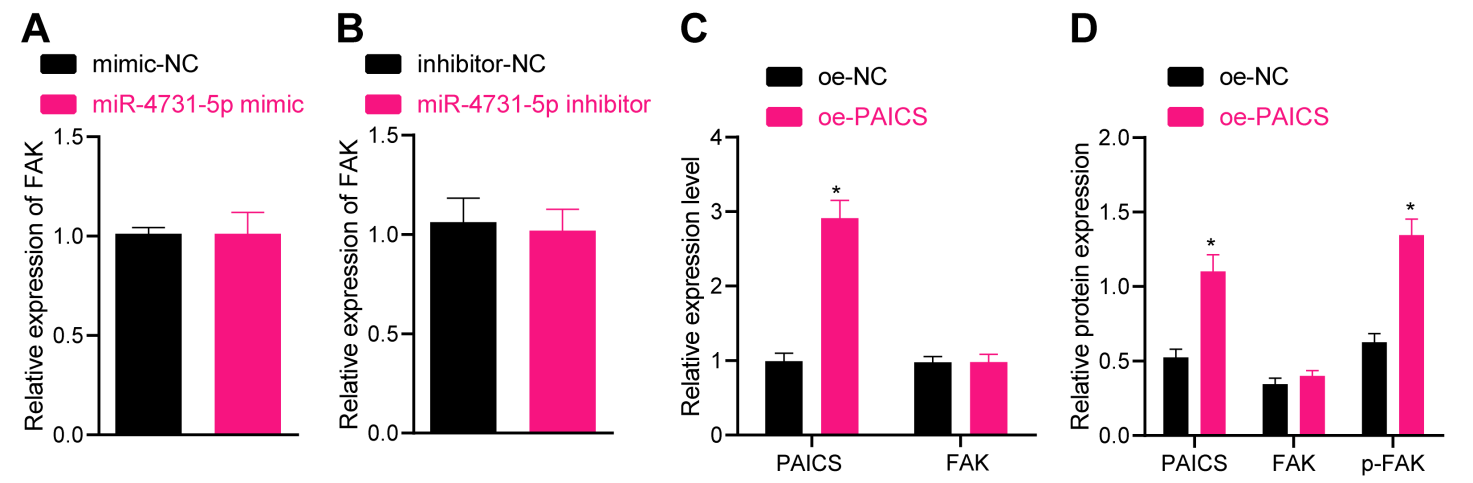
**

**Supplementary Fig. 6** PAICS induces phosphorylation of FAK in MDA-MB-231 cells. A, RT-qPCR was used to detect the expression of FAK after miR-4731-5p mimic treatment; B, Western blot analysis was used to detect the expression of FAK after miR-4731-5p inhibitor treatment; C, RT-qPCR was used to detect the expression of FAK after oe-PAICS treatment; D, Western blot analysis was used to detect the expression of PAICS, FAK and p-FAK after oe-PAICS treatment; Measurement data were expressed as mean ± standard deviation. Comparison between two groups was performed through independent *t*-test. * *p* < 0.05 compared with mimic NC, inhibitor-NC or oe-NC treatment.

**
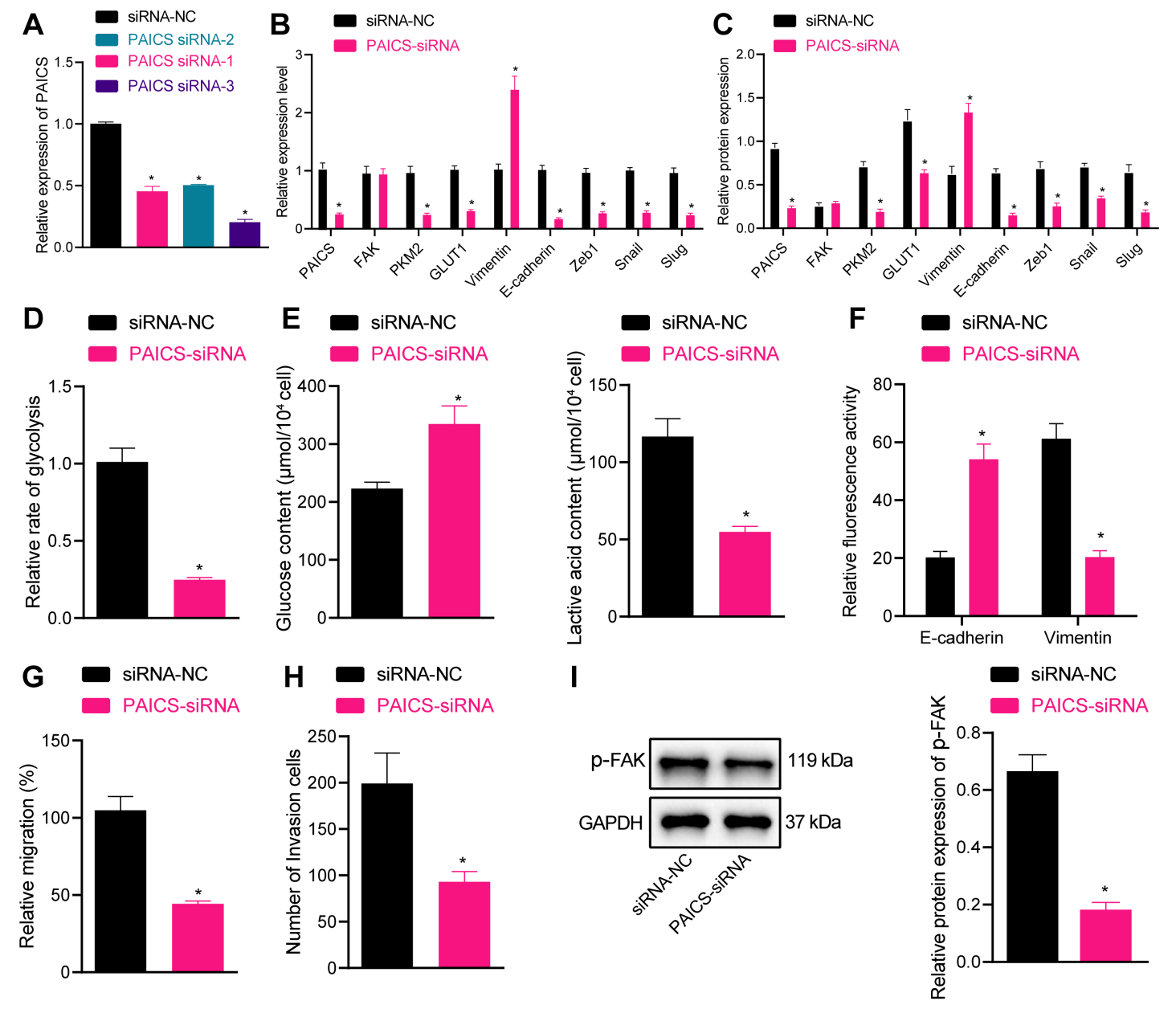
**

**Supplementary Fig. 7** Decreased PAICS inhibits the glycolysis, EMT and migration and invasion of MDA-MB-231 cells. A, RT-qPCR was used to detect the expression of PAICS in breast cancer cells; B, RT-qPCR was used to detect the expression of PAICS, FAK, PKM2, GLUT1, Vimentin, N-cadherin, Zeb1, Snail and Slug and E-Cadherin; C, Western blot analysis was used to detect the expression of PAICS, FAK, PKM2, GLUT1, Vimentin, N-cadherin, Zeb1, Snail and Slug and E-Cadherin; D, The effect of PAICS on glycolysis of breast cancer cells; E, The effect of PAICS on glucose and lactic acid content of breast cancer cells; F, Immunofluorescence was used to detect the expression of EMT-related markers Vimentin and E-Cadherin; G, Detection of cell migration by scratch test after PAICS siRNA treatment; H, Detection of cell invasion by Transwell assay after PAICS siRNA treatment; I, Western blot analysis was used to detect the expression of p-FAK after PAICS siRNA treatment; Measurement data were expressed as mean ± standard deviation. Comparison between two groups was performed through independent *t*-test. Analysis among multiple groups was conducted by one-way ANOVA followed by Tukey’s post hoc test. * *p* < 0.05 compared with siRNA-NC treatment.

**
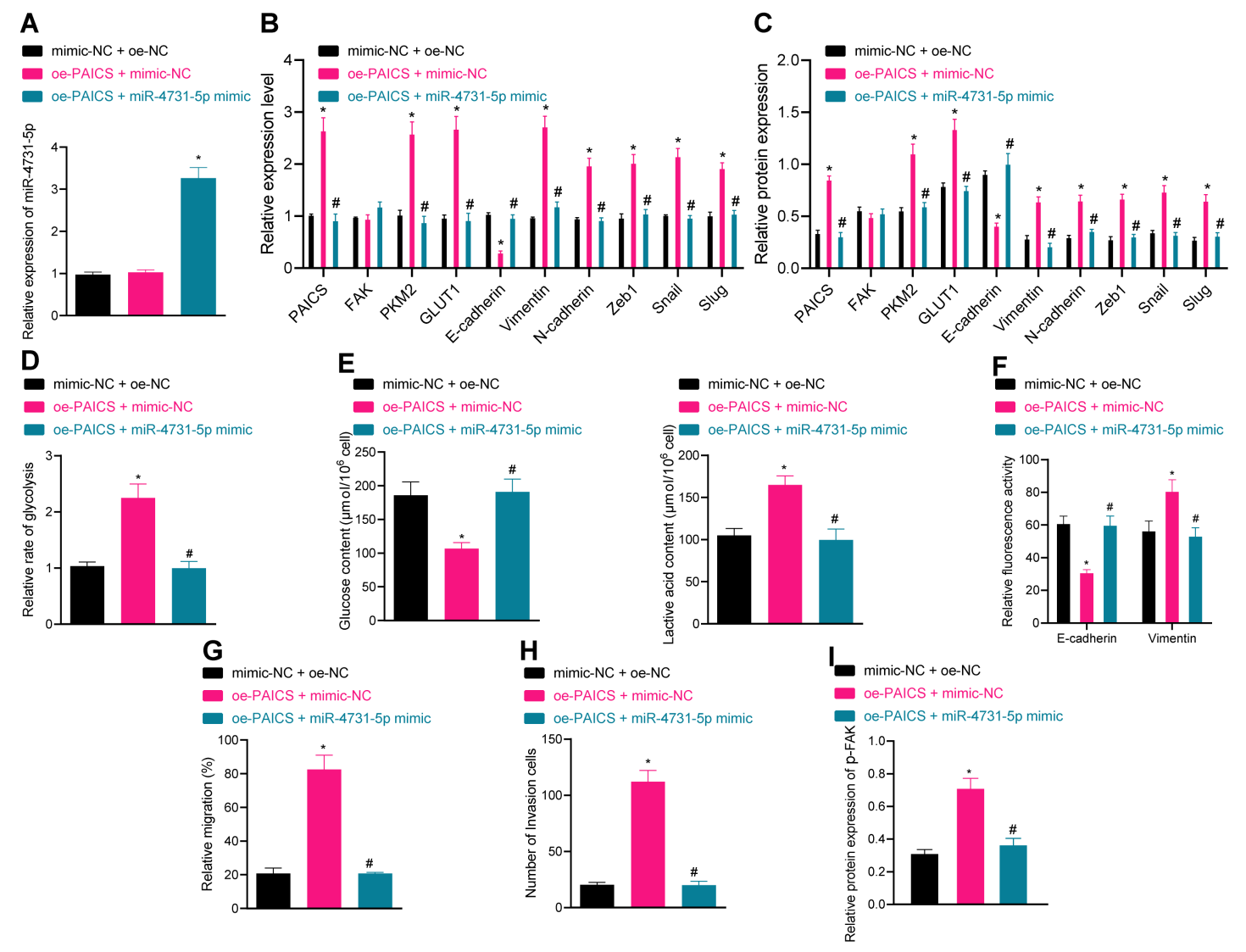
**

**Supplementary Fig. 8** miR-4731-5p inhibits the glycolysis and EMT of MDA-MB-231 cells through regulation of PAICS-mediated phosphorylation of FAK. A, RT-qPCR was used to detect the expression of miR-4731-5p in breast cancer cells after oe-PAICS and miR-4731-5p mimic treatment; B, RT-qPCR was used to detect the expression of PAICS, FAK, PKM2, GLUT1, Vimentin, N-cadherin, Zeb1, Snail and Slug and E-Cadherin in cells after oe-PAICS/miR-4731-5p mimic treatment; C, Western blot analysis was used to detect the expression of PAICS, p-FAK, PKM2, GLUT1, Vimentin, N-cadherin, Zeb1, Snail and Slug and E-Cadherin in cells after oe-PAICS/miR-4731-5p mimic treatment; D, The effect of oe-PAICS/miR-4731-5p mimic on glycolysis of breast cancer cells; E, The effect of oe-PAICS/miR-4731-5p mimic on glucose and lactic acid content of breast cancer cells; F, Immunofluorescence was used to detect the expression of EMT-related markers Vimentin and E-Cadherin; G, Detection of cell migration by scratch test after oe-PAICS/miR-4731-5p mimic treatment; H, Detection of cell invasion by Transwell assay after oe-PAICS/miR-4731-5p mimic treatment; I, Western blot analysis was used to detect the expression of p-FAK after oe-PAICS/miR-4731-5p mimic treatment; Measurement data were expressed as mean ± standard deviation. Comparison between two groups was performed through independent *t*-test. Analysis among multiple groups was conducted by one-way ANOVA followed by Tukey’s post hoc test. * *p* < 0.05 compared with mimic NC + oe-NC treatment. # *p* < 0.05 compared with oe-PAICS + mimic NC treatment.

**
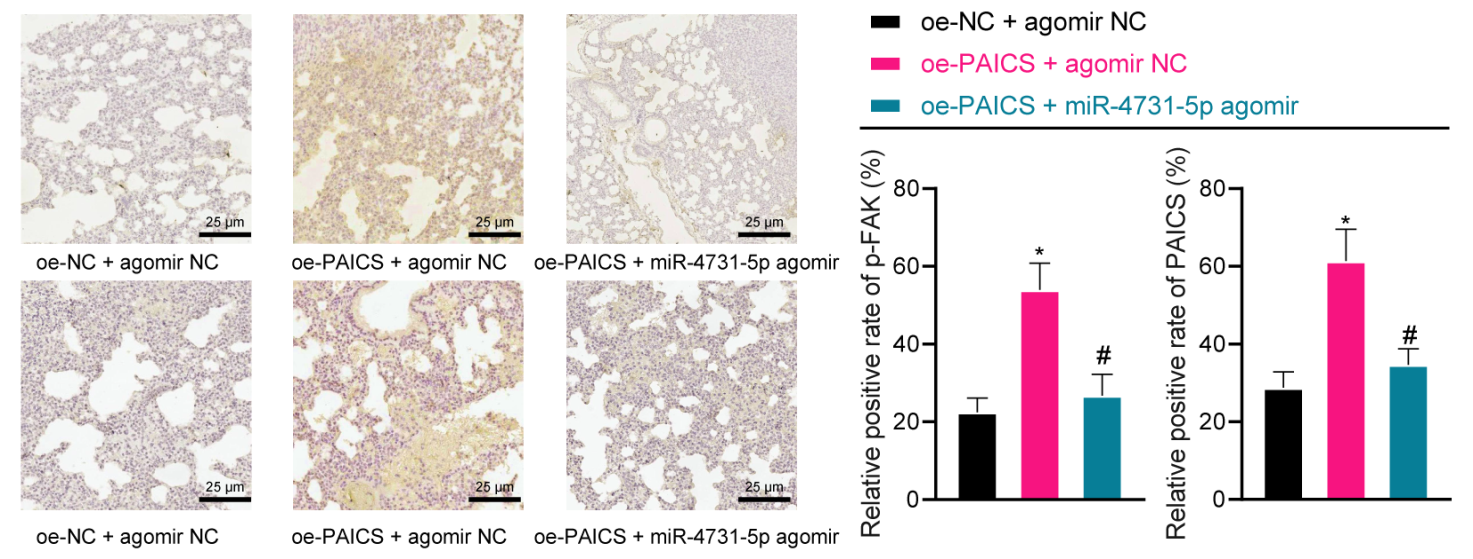
**

**Supplementary Fig. 9** The expression of p-FAK and PAICS in the breast cancer lung metastasis model determined by immunohistochemistry. , scale bar = 25 μm.
